# Platelet-rich fibrin ensures hemostasis after single-tooth removal under factor Xa inhibitors — a clinical prospective randomized split-mouth study

**DOI:** 10.1007/s00784-023-05317-3

**Published:** 2023-10-21

**Authors:** Solomiya Kyyak, Ali Jari, Diana Heimes, Julia Heider, Peer W. Kämmerer

**Affiliations:** Department of Oral and Maxillofacial Surgery, University Medical Center Mainz, Augustusplatz 2, 55131 Mainz, Germany

**Keywords:** Factor Xa inhibitors, Tooth extraction, Intra- and postoperative bleeding, Platelet-rich fibrin, Hemostatic effect, Hemostatic sponge

## Abstract

**Objectives:**

In this prospective, double-blinded, randomized split-mouth study, the local hemostatic effect of platelet-rich fibrin (PRF) inserted into the extraction socket in patients taking factor Xa (FXa) inhibitors (apixaban, rivaroxaban, edoxaban) was compared to a hemostatic gelatine sponge (GS) as the “therapeutic gold standard” without withdrawal of oral anticoagulant therapy.

**Materials and methods:**

Single-tooth extraction was conducted under local anesthesia in *n* = 21 patients using a split-mouth design (42 teeth). Using a double-blind approach, the extraction socket on one side of the jaw was filled with PRF and on the other with a GS. Bleeding was assessed immediately after surgery, in 30 min, 1 h, 1.5 h, and on follow-up appointments in 24 h and on the 7th day.

**Results:**

In 67% of cases, mild postoperative oozing could be stopped 30–90 min after tooth extraction via gauze pressure without any delayed bleeding. Concerning bleeding events, there was no difference among the PRF and GS groups and no significant difference among rivaroxaban, apixaban, and edoxaban (all *p* > 0.15).

**Conclusion:**

PRF and GS are reliable hemostatic methods in postextraction sockets of patients taking FXa inhibitors.

**Clinical relevance:**

Consequently, there is no need to discontinue FXa inhibitors because of a single-tooth removal, eliminating the risk of thrombus formation.

## Introduction

Patients with drug-induced bleeding disorders need special attention in oral and maxillofacial surgery [1]. Even after tooth extraction, patients taking antiplatelets or anticoagulants can experience life-threatening hemorrhage [2]. For prevention, suggested measures are local wound management, including hemostatic agents and/or interruption or reduction of antiplatelet/anticoagulant therapy, replacement of this treatment by bridging, or continuation of the medication [3–5]. Consequently, continuing anticoagulants increases the risk of bleeding, and withdrawal increases the risk of thrombus formation [4, 6].

Since most anticoagulants’ use is limited because of parenteral administration, frequent monitoring, and dose adjustments, novel small-molecule, oral, direct factor Xa (FXa) inhibitors were developed [7]. Because of its crucial role in the coagulation cascade and limited function outside coagulation, FXa has emerged as an attractive target for novel anticoagulants [8]. Thus, the new direct anticoagulants, as effective and safe as predecessors, showed minimal interference with food or other drugs and can be administered in fixed doses without routine coagulation monitoring [9, 10].

Nowadays, FXa inhibitors such as rivaroxaban, apixaban, and edoxaban are used for therapy and prevention of venous thromboembolism, stroke prevention in patients with atrial fibrillation [11, 12], and secondary prevention of acute coronary syndrome [3]. Patients are primarily older than 65, with decreased renal function and other comorbidities [13, 14]. Nevertheless, recent studies suggested that age, gender, and obesity do not have a clinically relevant effect on the pharmacokinetics and pharmacodynamics of FXa inhibitors [15, 16]. Furthermore, the influence of renal function on clearance of FXa inhibitors is moderate [17, 18], even in subjects with severe renal impairment (creatinine clearance < 30 mL/min) [19]. Over the years, the number of patients taking FXa inhibitors has been increasing tremendously [20, 21] with an estimated annual growth rate of 10% [22, 23]. Nevertheless, managing these patients in oral and maxillofacial surgery is for debate. First, FXa inhibitors provoke significant peri- and postoperative bleeding. Second, the risk of bleeding should be weighed against the risk of stroke or a potential thromboembolic event [24], and withdrawal of the medications should be avoided in most cases [4, 25]. For apixaban and rivaroxaban, an antidote (andexanet alfa) is available but not approved for surgical patients; it has a short duration and high costs [26]. Thus, local hemostatic measures and postoperative instructions of patients under anticoagulant therapy are essential [4, 5, 27]. Local measures include meticulous curettage and debridement, suturing, local compression via pressure [28], application of additional local hemostatic substances such as histoacrylic glue [29], tranexamic acid [6], hemostatic sponges, fibrin glue, oxidized cellulose [30, 31], surgical diathermy [32], and a prolonged period of post-interventional observation [4, 32].

Although hemostatic sponges are considered a gold standard for managing tooth socket bleeding, there is no proof of their efficiency after surgical procedures in the maxillofacial area in patients under FXa inhibitors [33]. For decades, platelet-rich fibrin (PRF) showed significant regenerative features in maxillofacial surgery [33–36]. Moreover, PRF is advantageous, being a complete autologous product without any additives. In addition, a specific hemostatic effect after dental-alveolar surgery was demonstrated [34–36] as far as PRF contains concentrates of fibrin and platelets, gained from centrifugated patient’s blood. Therefore, a simple tooth extraction model, a procedure with a low risk of bleeding [37], was chosen to examine the potential of PRF compared to a hemostatic sponge as a hemostatic inserted into the extraction socket in patients under FXa inhibitor therapy without withdrawal of the drug.

Thus, in this clinical prospective randomized, double-blinded split-mouth study, the local hemostatic effect of autologous PRF as inserted into the extraction teeth socket in patients taking direct FXa inhibitors is to be compared to the hemostatic gelatine sponge (GS) as the “therapeutic gold standard” without withdrawal of oral anticoagulant therapy. The study’s primary aim was to define the most reliable method for hemostasis after tooth extraction without withdrawing FXa inhibitors. Secondary outcome was if PRF controls bleeding in the early postoperative stages better than GS in the same cohort of patients.

## Materials and methods

### Study design and sample size calculation

In this clinical prospective randomized, double-blind study, single-tooth extractions were conducted in two contralateral sites of the same jaw using a split-mouth design in patients taking FXa inhibitors. The double-blinded approach was performed via sealed envelopes as described below. The extraction socket of the test group on one side of the jaw was filled with PRF and the one of the control group on the other side of the jaw with a hemostatic GS (Hygitec, Aegis Lifesciences, Gujarat, India). Ethical approval was obtained from the Ethical Review Board of Landesärztekammer Rheinland-Pfalz (Prot. No. 2021–16116 of 3rd May 2022).

### Study population

A cohort of patients of the Department of Oral and Maxillofacial Surgery of University Medical Center Mainz, Germany, were included in the study from May 2022 to December 2022. General inclusion criteria were people of any age or gender under monotherapy of FXa inhibitor without other additional antithrombotic or anticoagulant medication, with normal blood coagulation parameters and that required extraction of two teeth each on contralateral sides of the same jaw. Exclusion criteria were patients with hepatic dysfunction; hematologic diseases; thrombocytopenia; immune-, radio-, or chemotherapy; bisphosphonate and antiresorptive therapy; pregnant or nursing patients; uncompensated diabetes; patients with hypersensitivity or allergic reactions to the local anesthetic; disabled patients; a tooth involved in an acute inflammatory process; cyst or tumor; extraction tooth sides needing osteotomy (ankylosis or hypercementosis); need for subperiosteal preparation; contact of the extraction socket with the mandibular nerve canal; connection to the maxillary sinus after extraction.

All patients were informed and signed a consent form for inclusion in the study. All patients underwent presurgical orthopantomography and a postoperative clinical control immediately after the surgery, after 30 min, after 1 h, 1.5 h, and at follow-up appointments at 24 h, and on the 7th day after the tooth extraction.

### Surgical protocol

Patients were instructed to take their medication, including FXa inhibitors, as usual. After local infiltration anesthesia with 4% articaine + epinephrine 1:200,000 (Ultracain D-S, Septodont GmbH, Niederkassel, Germany), tooth extraction on both sides of a jaw and curettage of the alveolar sockets was conducted by an experienced surgeon (investigator 1). The following steps were done by a different surgeon (investigator 2) who was not involved in the tooth extraction and did not conduct the postoperative control. Investigator 2 opened the closed envelopes with instructions for randomization and inserted a PRF plug in one socket (test group) and a solely hemostatic GS (Hygitec, Aegis Lifesciences) into the other one.

**Fig. 1:**
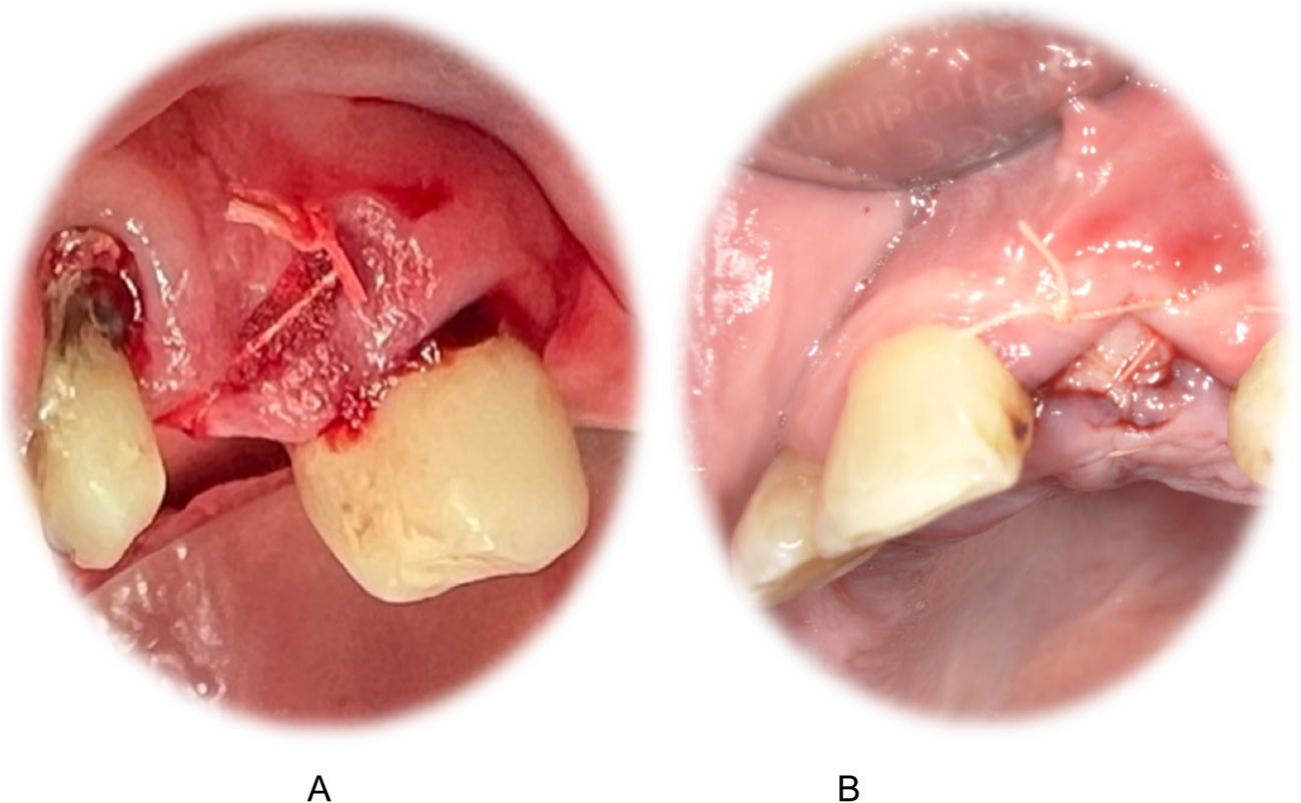
Postextraction alveolar socket filled with a GS (**A**) and PRF (**B**) as well as adaptive cross-mattress absorbable sutures were applied (**B**)

For PRF, 10 ml peripheral venous blood per matrix was collected from the patient after a cephalic or median cubital vein puncture. The vacutainer system and specific sterile plain vacuum tubes (A-PRF, Mectron, Carasco, Italy) were used. For centrifugation, a fixed angle rotor with a radius of 110 mm with 1200 rpm and a relative centrifugal force of 177 g for 8 min was employed (Duo centrifuge, Mectron, Carasco, Italy), according to the manufacturer’s instructions. Ten minutes after the centrifugation, fibrin clots were prepared using the piston and cylinder assembly for 5 min (Xpression™ Box, IntraSpin, Intra-Lock, FL, USA) [38]. Investigator 2 closed both sides with adaptive cross-mattress absorbable sutures (Vicryl 3/0, Ethicon, Edinburgh, UK; Fig. 1). Postoperative instructions included local cooling and analgesics (paracetamol 500 mg 1–1-1 for 24 h).

Bleeding was assessed immediately after the surgery, after 30 min, 1 h, 1.5 h, and on follow-up appointments at 24 h, and on the 7th day after the tooth extraction by investigator 1, who was blinded to the hemostatic material. The results on the day of extraction were interpreted as follows: no bleeding, bleeding which stopped after 30 min of gauze tamponade, bleeding which stopped after 1-h gauze tamponade, and bleeding which stopped after 1.5 h of gauze tamponade. Patients were instructed to use pressure with gauze tamponades in domestic bleeding events. In cases of unstoppable bleeding, patients were asked to return to the department. Bleeding events were classified into mild bleeding/oozing stopped via local pressure, moderate bleeding needing interventions than local pressure, and severe bleeding urgently needing interventions.

### Statistical analysis

After a sample size calculation using the parameter “postoperative bleeding” via split-mouth design [39] and corresponding to a similar study [31], 42 sites (*n* = 21 test groups and *n* = 21 control groups) and 21 patients were included (two groups per patient). Data analysis considered the presence of comorbidities; additional predictors were age, gender, type of comorbidity, clearance indexes, smoking habits, indication for FXa inhibitor therapy, FXa inhibitor agent, the interval between extraction, and the last intake of FXa inhibitor. All data showed normal distribution. Thus, a two-sided Student’s *t*-test for paired samples (*t*-test) was employed. A Kruskal–Wallis rank sum test (KWT) was also applied to compare all groups, including correlations. A *p*-value of ≤ 0.05 was considered to be statistically significant. Due to low group sizes, subgroup analyses on different FXa inhibitors were conducted descriptively only.

## Results

### Patient characteristics

The mean age of patients was 71 ± 2.8 years, with a range of 45–89 years. Females and males were almost evenly distributed, with 11 and 10, respectively. Even distribution was observed among the mandible and maxilla and location of extracted teeth (side teeth: molars and premolars; front teeth: incisors and canine teeth). Three patients were smokers. The subjects with rivaroxaban accounted for nine persons; seven patients took apixaban, and five took edoxaban. Nine patients suffered from arrhythmia/atrial fibrillation, ten from deep vein thromboses, and two have had pulmonary embolism (Tables 1 and 2).

**Table 1:** Summary of the case series

	Amount	Percentage, %
Age (years)		
Mean ± SEM	71.04 ± 2.82	-
Range	45–89 years	-
Sex		
Male	10	52
Female	11	47
Medications		
Apixaban 2.5 mg**	5	33
Apixaban 5.0 mg**	2	
Rivaroxaban 10 mg*	4	43
Rivaroxaban 20 mg*	3	
Rivaroxaban 15 mg*	2	
Edoxaban 60 mg*	5	23
Indication		
Arrhythmia	9	42
Deep vein thrombosis	10	47
Pulmonary embolism	2	4

^*^Intake 1 time per day; **intake two times per day

**Table 2:** Summary of the case series as the correlation of ingestion time and extraction time as published before [57]

*N*	FXa inhibitor, dose (mg)	Preoperative time of ingestion	Time of exodontia	Postoperative time of ingestion	Teeth extracted	Postoperative bleeding (oozing) directly after tooth removal
1	Rivaroxaban 10	8:00	10:00	-	11/22	No
2	Apixaban 2.5	7:00	09:15	19:00	37/47	Stopped in 1.5 h
3	Rivaroxaban 10	8:00	09:00	-	23/13	No
4	Apixaban 2.5	7:00	13:00	19:00	35/44	Stopped in 1 h
5	Apixaban 5	8:00	10:00	20:00	44/34	Stopped in 30 min
6	Edoxaban 60	9:00	13:00	-	23/11	No
7	Rivaroxaban 20	8:00	09:15	-	13/23	Stopped in 30 min
8	Rivaroxaban 10	07:00	14:00	-	32/41	Stopped in 30 min
9	Edoxaban 60	07:00	11:00	-	44/34	Stopped in 30 min
10	Rivaroxaban 15	09:00	13:00	-	27/17	No
11	Rivaroxaban 20	07:00	08:15	-	23/12	Stopped in 30 min
12	Apixaban 2.5	08:00	15:00	20:00	22/12	No
13	Apixaban 2.5	07:00	14:30	19:00	16/26	No
14	Apixaban 2.5	07:00	13:00	19:00	32/41	Stopped in 30 min
15	Rivaroxaban 20	08:00	14:00	-	14/24	Stopped in 30 min
16	Rivaroxaban 15	09:00	10:00	-	47/36	Stopped in 30 min
17	Edoxaban 60	07:00	08:15	-	42/31	Stopped in 30 min
18	Edoxaban 60	07:00	09:30	-	22/12	Stopped in 30 min
19	Edoxaban 60	10:00	11:00	-	25/44	Stopped in 30 min
20	Apixaban 5	08:00	15:00	20:00	44/33	Stopped in 30 min
21	Rivaroxaban 10	09:00	13:00	-	35/45	No

### Bleeding events

Overall, only mild postoperative oozing (from 30 to 90 min after surgery) was observed, which could be stopped in most cases after 30 min with a maximum of 1.5 h after tooth extraction, and no hemorrhage was recorded. In brief, oozing events were seen in 28 cases. Here, in 24 cases, the oozing stopped after 30 min of gauze pressure on the wound; in two cases, the oozing stopped after 1 h of gauze pressure, and in two cases, after 1.5 h (Table 3). There was no difference between the test and control groups (all *p* > 0.05). Overall, there was no late postoperative bleeding later than 1.5 h after surgery (Table 3). No bleeding event was noticed after the suture removal on the 7th day.

**Table 3:** Summary of the bleeding/oozing events for all cases and for each group specifically (total* n* = 42)

Group	No bleeding	Oozing which stopped in 30 min	Oozing which stopped in 1 h	Oozing which stopped in 1.5 h	Postoperative bleeding after 1.5 h
PRF	7	12	1	1	0
Sponge	7	12	1	1	0

**Fig. 2:**
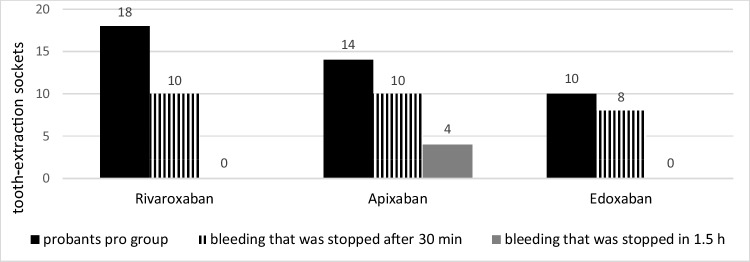
Group distribution according to the type of medication with its bleeding incidence (total* n* = 42)

**Fig. 3:**
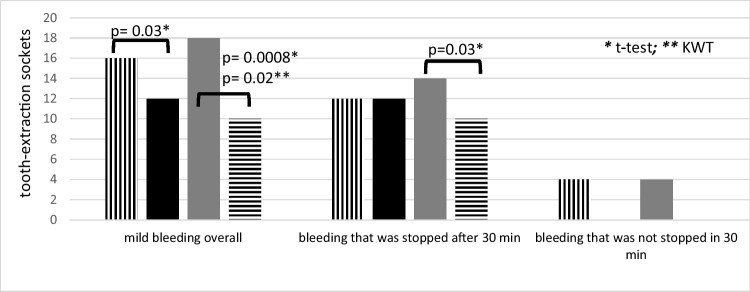
Bleeding event distribution in association with extraction sites (total* n* = 42)

**Fig. 4:**
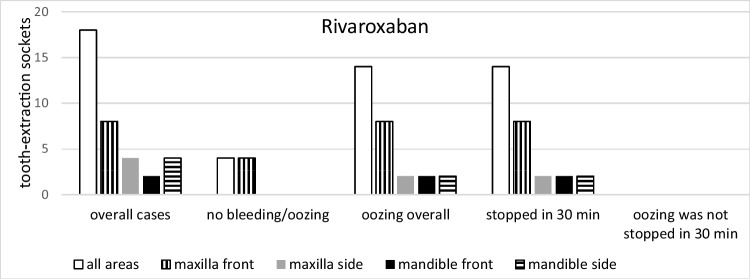
Group comparisons of the different drugs and the different sites (rivaroxaban*, n* = 18)

**Fig. 5:**
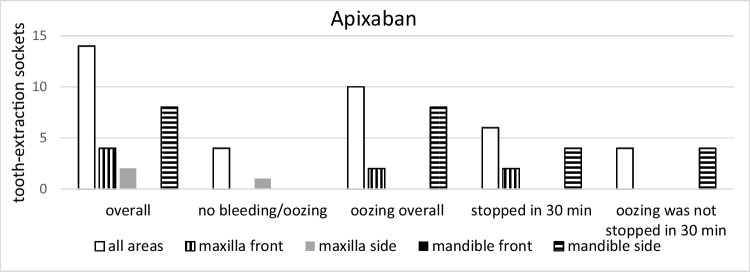
Group comparisons of the different drugs and the different sites (apixaban*, n* = 14)

**Fig. 6:**
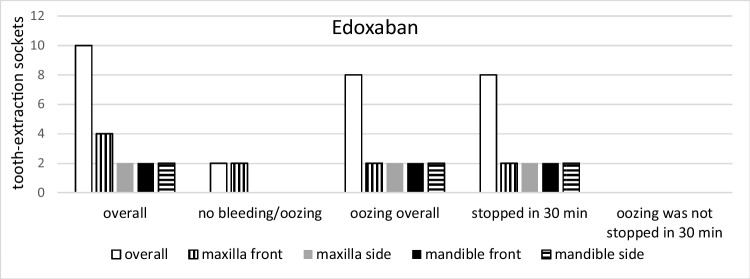
Group comparisons of the different drugs and the different sites (edoxaban*, n* = 10)

Specifically, bleeding events were observed for rivaroxaban, apixaban, and edoxaban in 18, 14, and 10 cases, respectively, however, without significant difference among the groups (*p* = 0.15* t*-test; *p* = 0.45 KWT). Bleeding in patients taking rivaroxaban and edoxaban could be stopped in all cases after 30 min of gauze pressure. In two patients taking apixaban, 1.5 h was needed for completed hemostasis (Figs. 2, 3, 4, 5, and 6).

There was a significant correlation between bleeding events and the location of the extracted teeth. In detail, significantly more bleeding events were seen in side teeth when compared to front teeth (16 and 12 cases, respectively; *p* = 0.03 *t*-test; *p* = 0.23 KWT) and in the mandible when compared to the maxilla (18 and 10 cases, respectively; *p* = 0.0008 *t*-test; *p* = 0.02 KWT) (Figs. 3, 4, 5, and 6). Oozing, which could be stopped after 1.5 h only, was exclusively seen in side mandibular teeth in patients under apixaban.

Patients taking higher doses of rivaroxaban (20 mg) and apixaban (5 mg) showed more postoperative oozing (Table 2). In the correlation between oozing and non-oozing events, edoxaban showed a ratio of 4:1, the highest of all present study groups (Fig. 6). The maximum creatinine level of included individuals was 1.28 mg/dl. The correlation between bleeding events and creatinine level was not observed; however, no statistical significance was calculated due to low patient numbers in subgroups.

Certain risk factors are correlated with prolonged postoperative bleeding events. In two cases with prolonged bleeding events, patients were older than 85 years. In one case, the patient showed slightly elevated renal values (creatinine: 1.13 mg/dl). In another case, the patient had smoking habits and a creatinine level of 1.28 mg/dl. Additionally, no correlation between the ingestion time of FXa inhibitors and bleeding events was found (*R*^2^ = 0.1875).

## Discussion

Generally, simple tooth extraction is interpreted as a procedure with a low risk of postoperative bleeding [3, 24]. Thus, it is not suggested to interrupt oral antithrombotic or anticoagulant medication, even dual antiplatelet therapy, in simple dental procedures [37]. According to German guidelines, apixaban, rivaroxaban, or edoxaban should not be stopped for uncomplicated oral surgery procedures. However, a detailed anamnesis of the patient is highly important [3, 40].

However, there is no evidence for oral surgery patients taking direct FXa inhibitors [41]. Thus, in the literature, mainly expert opinions and extrapolations are available [24]. Although there are numerous common clinical studies on bleeding events under dabigatran [21, 42, 43], it has distinctive pharmacokinetics and pharmacodynamics compared to apixaban, edoxaban, and rivaroxaban [35].

Rivaroxaban is the most commonly used FXa inhibitor. Generally, a minor postoperative bleeding risk of 4–7% in patients taking rivaroxaban is described, and significant bleeding events with subsequently decreased hemoglobin or need for transfusion account for only 1–2% [44]. However, these statistical statements do not correspond to findings in the present study focusing on minor oral surgery, where neither prolonged bleeding nor hemorrhage after tooth extraction was detected. It is proposed that by creatinine clearance of less than 29 ml/min, direct oral FXa inhibitors may reach a maximum concentration in plasma and longer half-life, indicating a more extended withdrawal period before surgery [45]. On the other hand, it is suggested that the pharmacokinetics of FXa inhibitors attenuate the risk of drug accumulation in patients with renal impairment [46]. This is because of the dual elimination of FXa inhibitors via renal and fecal routes [19, 47]. Nevertheless, no patients with severe renal impairment were included in the present study, and the maximum creatinine level of included individuals was 1.28 mg/dl. Besides, no correlation between bleeding events and (low) creatine levels was seen, and a low patient number in subgroups was available only.

In a literature review by Wahl et al., the authors suggested that all but 2% of bleeding events of all dental surgical procedures could be controlled by local hemostatic measures [48]. In the present study, postextraction sockets were managed according to the German guidelines using hemostatic sponge and adaptive sutures. Subsequently, the authors have observed only postoperative oozing, which could always be stopped with gauze pressure without evidence of hemorrhage or delayed bleeding. According to Kämmerer et al., hemostatic measures such as using a hemostatic sponge and postoperative instructions are essential for anticoagulant therapy [3]. A prospective study assessing the risk of hemorrhage in patients with antiplatelet therapy suggests that postoperative bleeding after minor surgical interventions could be managed similarly by healthy patients, namely, using a pressure pack for the first 30 min [32]. In case of prolonged bleeding events, Girotra et al. suggested the following hemostatic measurements: applying a pressure pack, suturing, local hemostatic agents, and surgical diathermy. Based on their study, the authors also described the following additional measures for decreasing the bleeding risk. In the case of granulation tissue, a detailed curettage and debridement should be applied; after the surgical procedure, 30 min of observation should be planned, and personal consulting of the surgeon with the patient’s physicians should be conducted [32]. Some prospective studies found no advantage in suturing [31, 49]. Another suggests that suturing in combination with local hemostatic medications is of significance [50]. Some authors assign that neither advantages nor disadvantages could be found [81–83] compared to the different individual measures.

Mourao et al. found a positive influence of PRF on extraction sockets in patients receiving anticoagulant therapy. The study, however, did not include information about early postoperative bleeding, and PRF was used in all patients without a control group [51]. The present analysis compared local hemostasis with PRF on one side and the hemostatic sponge on the other. Hence, there was no difference between the two groups concerning oozing events. However, one should mention that PRF collection causes additional minor morbidity for patients and needs additional preoperative time.

Interestingly, prolonged oozing was observed in patients over 85 years. That may indicate the clinical relevance of the patient’s age, probably due to minor kidney function or other age-related factors. However, a higher number of elderly patients should be included for reliable results. Besides, the present study detected a potential correlation between smoking habits and postoperative oozing. No oozing in younger smokers (< 60 years) and prolonged oozing ≥ 1.5 h in an elderly patient (89 years old) with a smoking habit was observed. Nevertheless, because of the small number of patients with smoking habits, the data cannot be reliable and further studies are required. Furthermore, a second control group without any hemostatic agent was not included as historical data shows that the bleeding without hemostatic measures is significantly higher in anticoagulated patients [3]. Therefore, at least oxidized cellulose or hemostatic sponges were used in most studies [52, 53]. Previous studies present that the bleeding risk among FXa inhibitors is similarly decreased in comparison to other anticoagulants; moreover, pharmacokinetics and pharmacodynamic of the abovementioned medications are common [54–56]. Subsequently it is eligible to combine all three FXa inhibitors into both main and control groups for statistical reliable results. With low patient number, still, data of subgroups was also compared, to raise the question of relevance for further studies.

## Conclusion

For extraction of non-adjacent teeth in patients under monotherapy with FXa inhibitors and without other bleeding disorders, there is no need to interrupt FXa inhibitors. In this case, adequate local hemostasis of the extraction socket may be necessary. Subsequently, PRF or a hemostatic sponge as inserts and adaptive sutures are acceptable for hemostasis of extraction sockets. A maximum of 1.5 h postoperative oozing may be expected, which can be controlled by gauze pressure on the wound. The aforementioned treatment can be safely carried out on an outpatient basis. Early postoperative control appointments may not be necessary. In addition, information about the last intake of FXa inhibitors before tooth extraction may not need to be addressed.

## Data Availability

The data that support the findings of this study are available on request from the corresponding author, SK.
